# Tobacco smoke condensate-induced senescence in endothelial cells was ameliorated by colchicine treatment via suppression of NF-κB and MAPKs P38 and ERK pathways activation

**DOI:** 10.1186/s12964-024-01594-x

**Published:** 2024-04-03

**Authors:** Dilaware Khan, Huakang Zhou, Jinliang You, Vera Annika Kaiser, Rajiv K Khajuria, Sajjad Muhammad

**Affiliations:** 1 Department of Neurosurgery, Medical Faculty, University Hospital Düsseldorf, Heinrich-Heine- Universität Düsseldorf, Moorenstr.5, Düsseldorf, 40225 Germany; 2 Department of Neurosurgery, University Hospital Helsinki, Topeliuksenkatu 5, Helsinki, 00260 Finland

**Keywords:** Tobacco smoke, HUVECs, NF-kB, MAPKs, Senescence, SASP factors

## Abstract

Smoking is the major cause of cardiovascular diseases and cancer. It induces oxidative stress, leading to DNA damage and cellular senescence. Senescent cells increase the expression and release of pro-inflammatory molecules and matrix metalloproteinase, which are known to play a vital role in the initiation and progression of cardiovascular diseases and metastasis in cancer. The current study investigated the smoking induced cellular senescence and employed colchicine that blocked senescence in endothelial cells exposed to tobacco smoke condensate. Colchicine prevented oxidative stress and DNA damage in tobacco smoke-condensate-treated endothelial cells. Colchicin reduced β-gal activity, improved Lamin B1, and attenuated cell growth arrest markers P21 and P53. Colchicine also ameliorated the expression of SASP factors and inhibited the activation of NF-kB and MAPKs P38 and ERK. In summary, colchicine inhibited tobacco smoke condensate-induced senescence in endothelial cells by blocking the activation of NF-kB and MAPKs P38 and ERK.

**Graphical Abstract:**

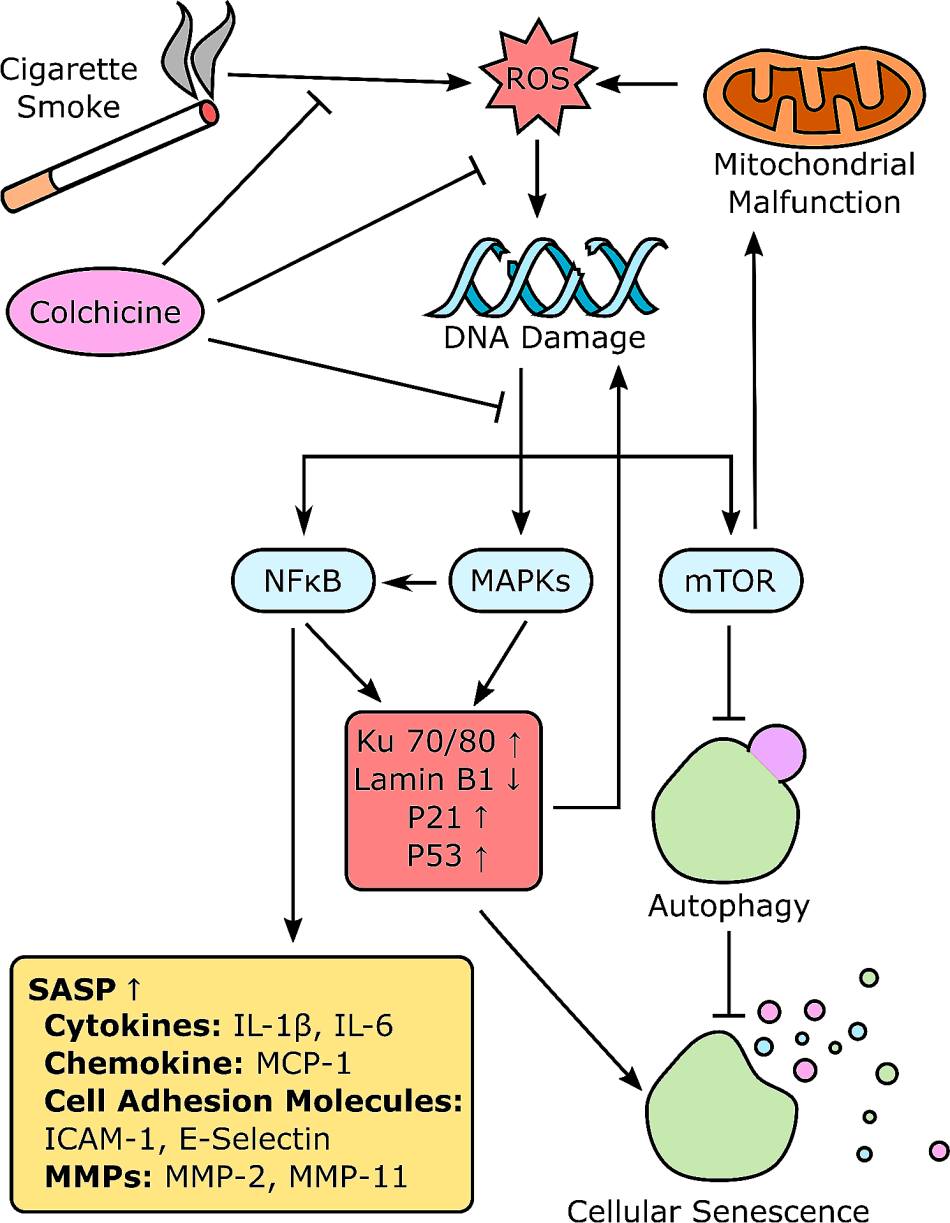

**Supplementary Information:**

The online version contains supplementary material available at 10.1186/s12964-024-01594-x.

## Introduction

Tobacco smoking is one of the major preventable causes of premature deaths globally and is a potentially acquired risk factor for cardiovascular diseases (CVDs), chronic obstructive pulmonary disease, and cancer [1–3]. The scientific literature suggests tobacco smoking to be a causative agent for CVDs and cancers [2]. According to the World Health Organization, tobacco kills more than 8 million people each year worldwide, including around 1.3 million people who do not smoke but are exposed to second-hand smoke.

There are more than 7000 chemicals in cigarette smoke, of which 250 are harmful, and 69 are well-established carcinogens [3, 4]. The constituents of cigarette smoke generate oxidative stress, which can subsequently cause DNA damage, leading to stress-induced premature cellular senescence [4–6]. Cellular senescence is characterized by cell cycle arrest, macromolecular damage, metabolic dysfunction, and a shift in the expression of secondary markers [7, 8]. Transient cellular senescence can exert a beneficial impact, such as wound healing and tumor suppression [6, 9]. However, the chronic accumulation of senescent cells can impair wound healing, accelerate aging, promote inflammation, and give rise to chronic diseases such as CVDs, cancer, and neurodegenerative diseases [9–11]. In healthy and young individuals, immune cells eliminate senescent cells [10, 12]. However, age-dependent deregulation of the immune system and immunosenescence can decrease the removal of senescent cells by immune cells [12]. Additionally, smoking can negatively influence the phagocytic activity of immune cells [13]. Consequently, senescent cells accumulate in different tissues, resulting in enhanced tissue senescent cell burden [12]. The data from human samples and experimental animal models implicate senescent in age-related diseases like atherosclerosis, abdominal aortic aneurysm, arterial stiffness, hypertension, and heart failure [14–16]. The selective removal of senescent cells reduced the disease burden and increased the life span in animal models [6, 10, 14–16].

In addition to that, senescent cells remain metabolically active and switch to a pro-inflammatory state, known as senescence-associated secretory phenotype (SASP) [6, 15]. The enhanced expression and release of SASP factors, including cytokines, chemokines, cell adhesion molecules, and matrix metalloproteinase (MMPs), promote and facilitate the recruitment, infiltration, and accumulation of immune cells in tissue [15, 17]. The SASP factors released from senescent cells induce and reinforce senescence in the neighboring cells, including infiltrated immune cells [6, 11]. Senescence of immune cells results in decreased phagocytosis, altered cytokine production, impaired antigen presentation, delayed resolution of inflammation and injury, and impaired chemotaxis [12]. All these mechanisms working together result in the formation of a chronic sterile pro-inflammatory microenvironment that can promote the initiation and progression of age-related diseases, including cardiovascular diseases (CVD) and cancer [11, 15, 17]. Furthermore, MMPs via tissue remodeling and post-translation processing of SASP factors contribute to the initiation and progression of CVD and metastasis in cancer [18]. Clinical and experimental studies have reported a strong positive correlation between enhanced expression of SASP factors and exacerbation of CVDs and cancers [11, 15]. Previously, using different cellular and animal disease models, colleagues have shown that blocking the expression and release of SASP factors could delay and prevent the initiation and progression of CVDs and cancers [11, 19, 20].

Oxidative stress and oxidative stress-induced DNA damage caused by smoking can activate NF-κB, MAPKS, and mTOR pathway [21]. These pathways are implicated in cellular senescence [21]. The transcription of SASP factors is regulated by NF-κB-P38MAPK signaling, and protein translation is regulated by mTOR [15]. Blocking the activation of these pathways inhibits senescence and the release of SASP factors [6, 15]. Moreover, the activation of these pathways has been associated with CVDs and cancer [17, 19]. Colchicine has been used for medicinal purposes since the dawn of time. In vitro and in vivo studies have shown the anti-inflammatory and antioxidant properties of colchicine [22–27]. Clinical trials and experimental animal studies reported the beneficial effects of colchicine against CVDs [22, 23, 25, 28, 29].

This study used colchicine to block senescence in endothelial cells exposed to tobacco smoke condensate. To understand the underlying mechanism, we investigated the impact of colchicine on the activation of NF-κB, MAPKs, and mTOR pathways in smoke-condensate-treated endothelial cells.

## Results

### Colchicine subdued smoke condensate-induced oxidative stress and DNA damage

**Fig. 1:**
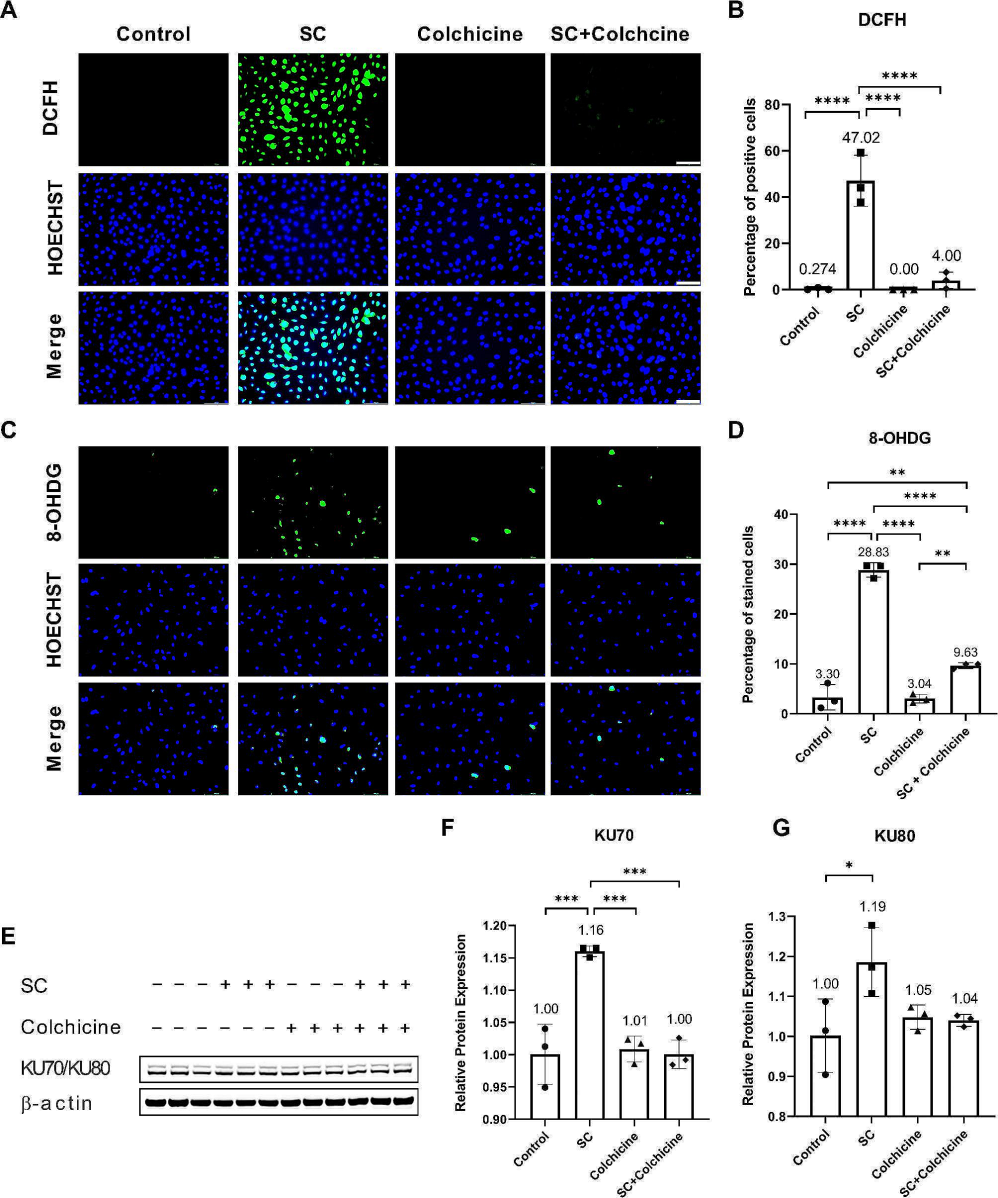
Colchicine prevented oxidative stress and oxidative stress-induced DNA damage in endothelial cells treated with smoke condensate. (**A**) DCFH-DA staining and (**B**) quantification of DCFH-DA staining. (**C**) Immunofluorescence (IF) staining for 8-OHDG and (**D**) quantification of 8-OHDG. (**E**) WB showing protein expression of KU70/KU80. Quantification of relative protein expression of (**F**) KU70 and (**G**) KU80. All experiments were performed in triplicates. β-actin was used as a control. SC = tobacco smoke condensate, *p < 0.05, **p < 0.01, ***p < 0.001, ****p < 0.0001, scale bar = 100 μm

Smoking causes oxidative stress [4, 5], which can induce DNA damage [6, 27]. To investigate whether colchicine can alleviate the effects of smoking on oxidative stress and oxidative stress-induced DNA damage, we treated endothelial cells with tobacco smoke condensate (50 µg/mL), colchicine (50 nM), and tobacco smoke condensate (50 µg/mL) combined with colchicine (50 nM) for 2 h. The untreated cells were used as a control. DCFH-DA staining revealed increased oxidative stress in smoke-condensate-treated endothelial cells (Percentage of positive cells: Control = 0.27 ± 0.48, SC = 47.02 ± 10.99, Colchicine = 0.00 ± 0.00, SC + Colchicine = 4.00 ± 3.54, *n* = 3, ****p < 0.0001, Fig. 1A, B). Colchicine ameliorated oxidative stress in endothelial cells exposed to smoke condensate (Fig. 1A, B). Immunofluorescence staining (IF) for oxidative stress-induced DNA damage marker 8-OHDG indicated increased oxidative stress-induced DNA damage in tobacco smoke-condensate-treated endothelial cells (Percentage of positive cells: Control = 3.30 ± 2.56, SC = 28.83 ± 1.45, Colchicine = 3.04 ± 0.88, SC + Colchcine = 9.63 ± 0.57, *n* = 3, **p < 0.01, ****p < 0.0001, Fig. 1C, D). Colchicine mitigated 8-OHDG expression in tobacco smoke-condensate-treated endothelial cells (Fig. 1C, D), suggesting that colchicine reduced oxidative stress-induced DNA damage in endothelial cells exposed to smoke condensate. Next, we performed protein analysis by Western blot (WB) for DNA repair proteins KU70/KU80. Interestingly, 24 h treatment with tobacco smoke-condensate increased the relative protein expression of DNA repair proteins KU70 and KU80 (Fig. 1E, F, G, and Table 1). Colchicine reduced the relative protein expression of KU70 in smoke-condensate-treated endothelial cells (Fig. 1E, F, and Table 1).

**Table 1:** Relative protein expression. The data were analyzed using one-way ANOVA followed by tukey’s test. SC = smoke condensate

Protein	Control	SC	Colchicine	SC + Colchicine	*p*-value
KU70	1.00 ± 0.05	1.16 ± 0.00	1.01 ± 0.02	1.00 ± 0.02	0.0003
KU80	1.00 ± 0.09	1.19 ± 0.09	1.05 ± 0.03	1.04 ± 0.01	0.0382
Lamin B1	1.00 ± 0.14	0.76 ± 0.00	1.06 ± 0.05	1.09 ± 0.09	0.0046
P53	1.00 ± 0.21	2.03 ± 0.13	1.06 ± 0.28	0.75 ± 0.39	0.0019
P21	1.00 ± 0.10	1.86 ± 0.35	1.45 ± 0.09	0.70 ± 0.22	0.0009
MMP-2	1.00 ± 0.05	1.60 ± 0.02	1.37 ± 0.15	1.20 ± 0.11	0.0004
P65	1.00 ± 0.08	1.00 ± 0.05	0.96 ± 0.03	0.93 ± 0.00	0.2722
p-P65	1.00 ± 0.09	1.51 ± 0.09	0.91 ± 0.03	1.11 ± 0.01	0.0001
p-65/P65	1.00 ± 0.05	1.51 ± 0.05	0.96 ± 0.06	1.20 ± 0.01	0.0001
p-P38	1.00 ± 0.14	5.76 ± 0.29	0.94 ± 0.02	1.03 ± 0.06	0.0001
p-ERK	1.00 ± 0.04	1.90 ± 0.24	1.08 ± 0.10	0.72 ± 0.08	0.0001
p-JNK	100 ± 0.18	1.08 ± 0.14	0.95 ± 0.12	1.10 ± 0.05	0.5054
p-mTOR	1.00 ± 0.18	1.21 ± 0.11	1.18 ± 0.11	1.06 ± 0.14	0.2772
p-S6	1.00 ± 0.03	1.69 ± 0.03	1.52 ± 0.03	1.22 ± 0.09	0.0001
p-4EBP1	1.00 ± 0.10	0.94 ± 0.24	1.40 ± 0.04	1.52 ± 0.19	0.0057

### Colchicine inhibited senescence in endothelial cells exposed to smoke condensate

**Fig. 2:**
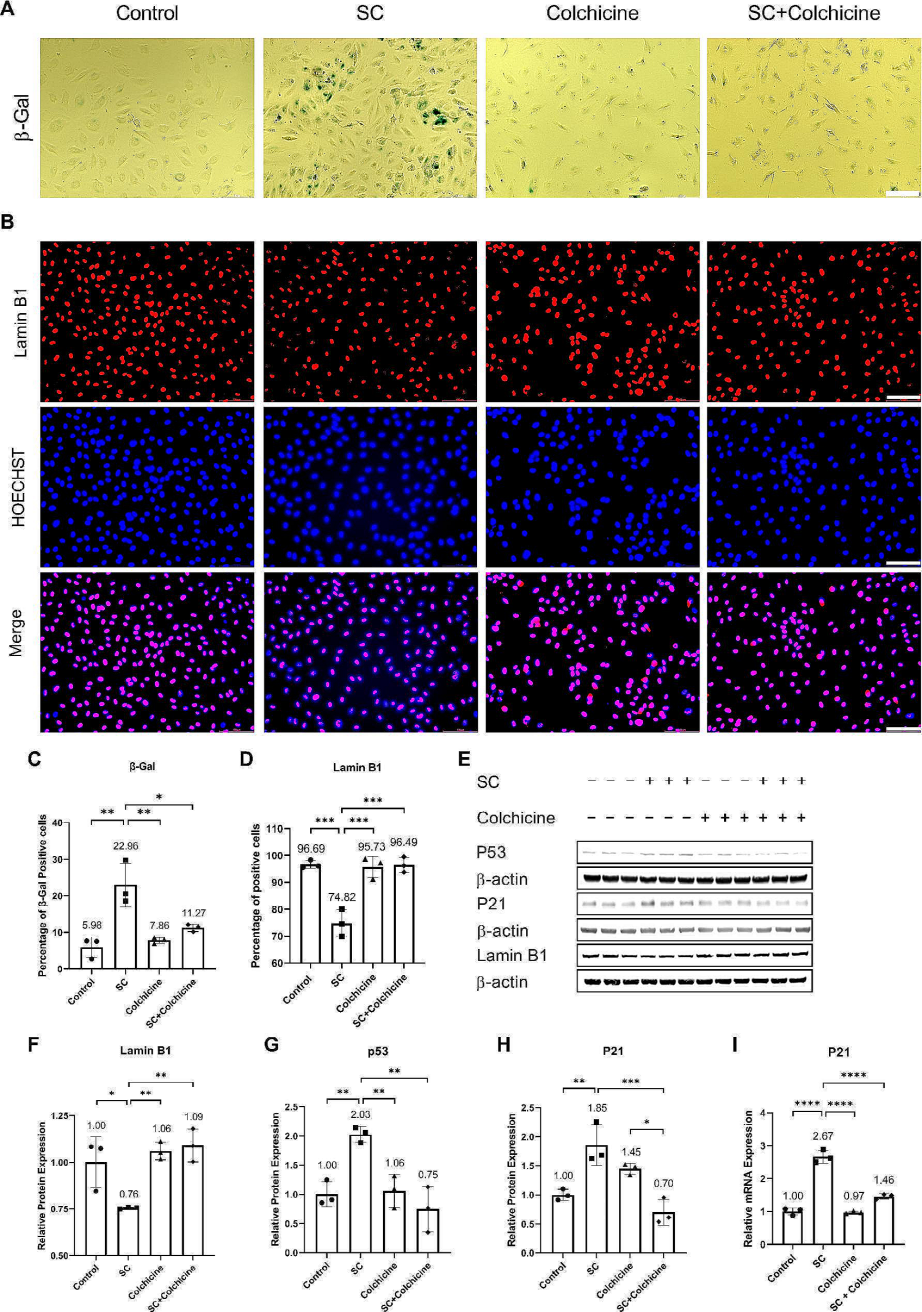
Colchicine inhibited smoke condensate-induced senescence in endothelial cells. (**A**) SA-β-gal staining and (**B**) IF staining for Lamin B1 in endothelial cells treated with different conditions for 24 h. The quantification of (**C**) SA-β-gal staining and (**D**) Lamin B1 IF staining. (**E**) WB showing protein expression of Lamin B1, P53, and P21. The relative protein expression of (**F**) Lamin B1, (**G**) P53, and (**H**) P21. (**I**) The relative mRNA expression of P21. The experiments were performed with biological triplicates. β-actin was used as a control. SC (smoke condensate). *p < 0.05, **p < 0.01, ***p < 0.001, ****p < 0.0001, scale bar = 100 μm

Oxidative stress and oxidative stress-induced DNA damage can cause cellular senescence. Previously, it has been recommended to validate cellular senescence using three characteristics [7, 8]. One of these features is a structural change in senescent cells, which can be confirmed by increased senescence-associated beta-galactosidase (SA-β-gal) activity and Lamin B1 loss [7, 8]. The endothelial cells were treated with different conditions for 24 h. The treatment with smoke condensate for 24 h increased β-gal activity (Percentage of positive cells: Control = 5.98 ± 2.81, SC = 22.96 ± 5.97, Colchicine = 7.86 ± 0.80, SC + Colchicine = 11.27 ± 1.03, *n* = 3, *p < 0.05, **p < 0.01, Fig. 2A, C), and the cells exhibited Lamin B1 loss (Percentage of positive cells: Control = 96.69 ± 1.47, SC = 74.82 ± 5.06, Colchicine = 95.73 ± 3.97, SC + Colchicine = 96.49 ± 2.82, *n* = 3, ***p < 0.001, Fig. 2B, D). Colchicine significantly reduced SA-β-gal activity (Fig. 2A, C) and restored Lamin B1 expression (Fig. 2B, D, E, and F; Table 1) in endothelial cells treated with smoke condensate.

The second attribute of senescent cells is cell growth arrest, which can be validated by increased expression of P53 and P21 [7, 8]. Compared to the control, the relative protein expression of P53 and P21 was elevated in smoke-condensate-treated endothelial cells (Fig. 2). Colchicine ameliorated the relative protein expression of P53 (Fig. 2G; Table 1) and P21 (Fig. 2H; Table 1). The mRNA analysis showed that P21 expression was regulated at the transcription level (Fig. 2I; Table 2).

**Table 2:** Relative mRNA expression. The data were analyzed using one-way ANOVA followed by tukey’s test. SC = smoke condensate

Gene	Control	SC	Colchicine	SC + Colchicine	*p*-value
P21	1.00 ± 0.11	2.67 ± 0.20	0.97 ± 0.03	1.46 ± 0.08	0.0001
TNF-α	1.00 ± 0.13	1.68 ± 0.08	1.56 ± 0.12	1.26 ± 012	0.0004
IL-1β	1.00 ± 0.04	1.60 ± 0.31	1.18 ± 0.06	1.12 ± 0.25	0.0305
IL-6	1.00 ± 0.02	4.76 ± 1.12	0.98 ± 0.16	2.20 ± 0.27	0.0001
IL-8	1.00 ± 0.02	1.28 ± 0.20	1.15 ± 0.07	2.36 ± 0.09	0.0001
MCP-1	1.00 ± 0.04	1.38 ± 0.21	0.84 ± 0.11	0.06 ± 0.02	0.0001
ICAM-1	1.00 ± 0.05	8.16 ± 0.64	2.42 ± 0.22	4.38 ± 0.85	0.0001
E-Selectin	1.00 ± 0.01	1.56 ± 0.20	0.79 ± 0.02	0.86 ± 0.09	0.0001
VCAM-1	1.02 ± 0.22	1.25 ± 0.16	1.12 ± 0.12	1.08 ± 0.28	0.5901
MMP-1	1.00 ± 0.09	30.74 ± 3.37	2.22 ± 0.14	36.02 ± 6.11	0.0001
MMP-2	1.01 ± 0.13	1.79 ± 0.17	0.90 ± 0.12	0.98 ± 0.10	0.0001
MMP-8	1.02 ± 0.25	2.32 ± 0.11	1.19 ± 0.04	2.35 ± 0.14	0.0001
MMP-10	1.01 ± 0.16	4.65 ± 0.61	5.66 ± 0.40	9.35 ± 0.88	0.0001
MMP-11	1.00 ± 0.02	1.95 ± 0.14	0.82 ± 0.07	0.81 ± 0.12	0.0001
TIMP-1	1.00 ± 0.07	1.14 ± 0.09	0.68 ± 0.11	0.33 ± 0.21	0.0003
TIMP-2	1.00 ± 0.08	2.39 ± 0.17	1.32 ± 0.10	2.10 ± 0.09	0.0001

**Fig. 3:**
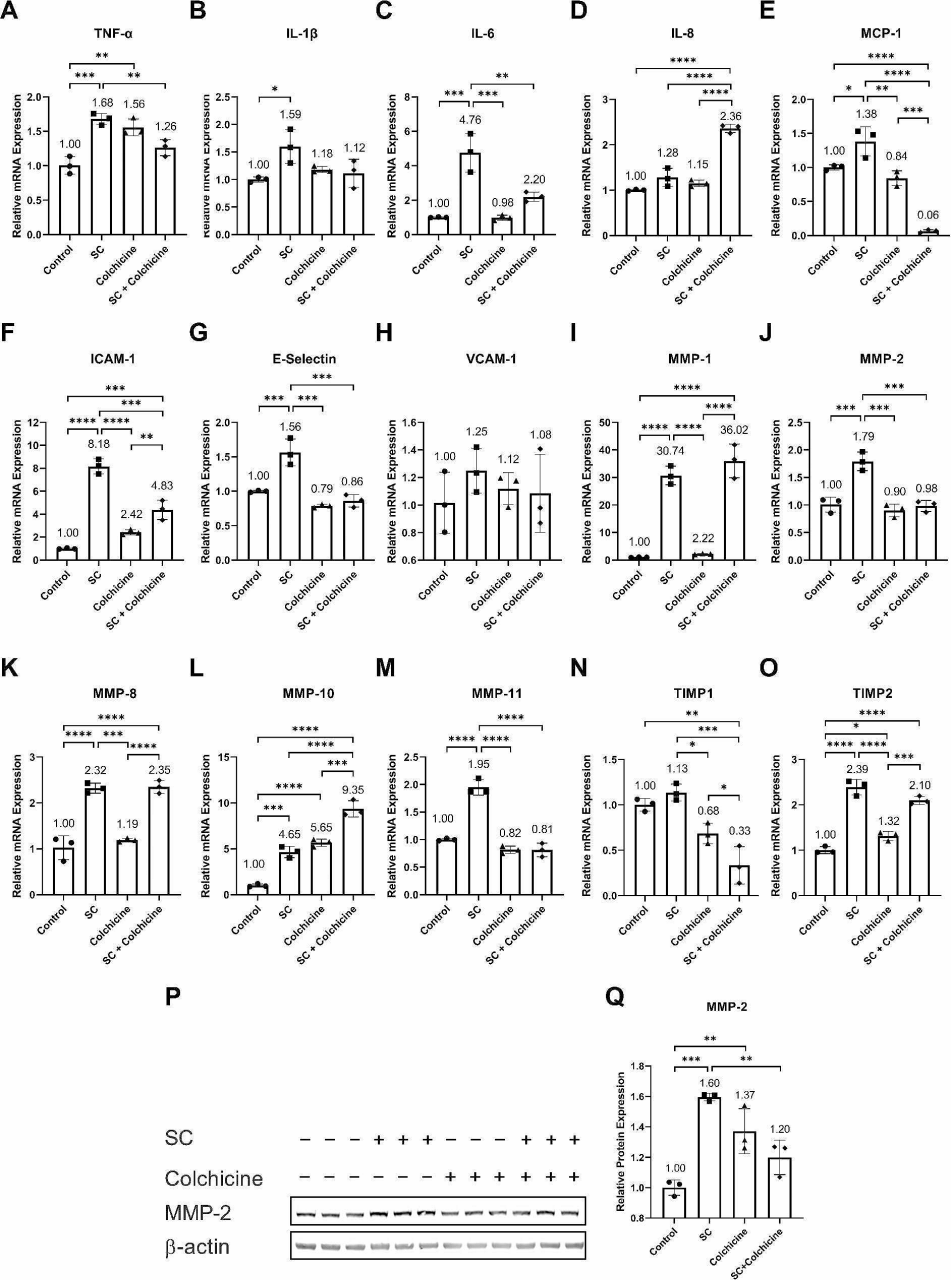
Colchicine suppressed the transcription of SASP factors. The relative mRNA expression of (**A**) TNF-alpha, (**B**) IL-1b, (**C**) IL-6, (**D**) IL-8, (**E**) MCP-1, (**F**) ICAM-1 (**G**) E-Selectin, (**H**) VCAM-1, (**I**) MMP1, (**J**) MMP2, (**K**) MMP8, (**L**) MMP10, (**M**) MMP11, (**N**) TIMP1, (**O**) TIMP2. (**P**) WB showing protein expression of MMP2. (**Q**) Quantified Relative protein expression of MMP2. The experiments were performed with biological triplicates. β-actin was used as a control. *p < 0.05, **p < 0.01, ***p < 0.001, ****p < 0.0001

Senescent cells exhibit higher expression and release of SASP factors [7, 8, 15]. Because colchicine diminished senescence-associated structural changes (Fig. 2A, B, C, D, F) and decreased cell growth arrest (Fig. 2G, H, I) in endothelial cells exposed to tobacco smoke condensate, we performed qPCR to investigate the impact of colchicine on the transcription of SASP factors in endothelial cells treated with tobacco smoke condensate. Tobacco smoke condensate induced the relative mRNA expression of SASP factors (Fig. 3; Table 2). Colchicine mitigated the relative mRNA expression of TNF-alpha, IL-6, MCP-1, ICAM-1, E-Selectin, MMP-2, and MMP-11 in endothelial cells treated with tobacco smoke condensate (Fig. 3A, C, E, F, G, J, M; Table 2). The relative mRNA expression of IL-8 was significantly higher in endothelial cells exposed to the combined treatment of tobacco smoke condensate and colchicine than in the controls and the endothelial cells treated with either tobacco smoke condensate or colchicine alone (Fig. 3D; Table 2). Neither treatment affected the relative mRNA expression of VCAM-1 in endothelial cells (Fig. 3H; Table 2). Colchicine could not reduce the relative mRNA expression of IL-1b, MMP-1, MMP-8, and TIMP-2 (Fig. 3B, I, K, O; Table 2). Tobacco smoke condensate and colchicine increased the relative mRNA expression of MMP-10 as compared to the control, and the combination of tobacco smoke condensate and colchicine increased synergically MMP-10 transcription (Fig. 3L; Table 2). Colchicine also attenuated the relative protein expression of MMP-2 in tobacco smoke-condensate-treated endothelial cells (Fig. 3P, Q; Table 1).

### Pathway analysis

**Fig. 4:**
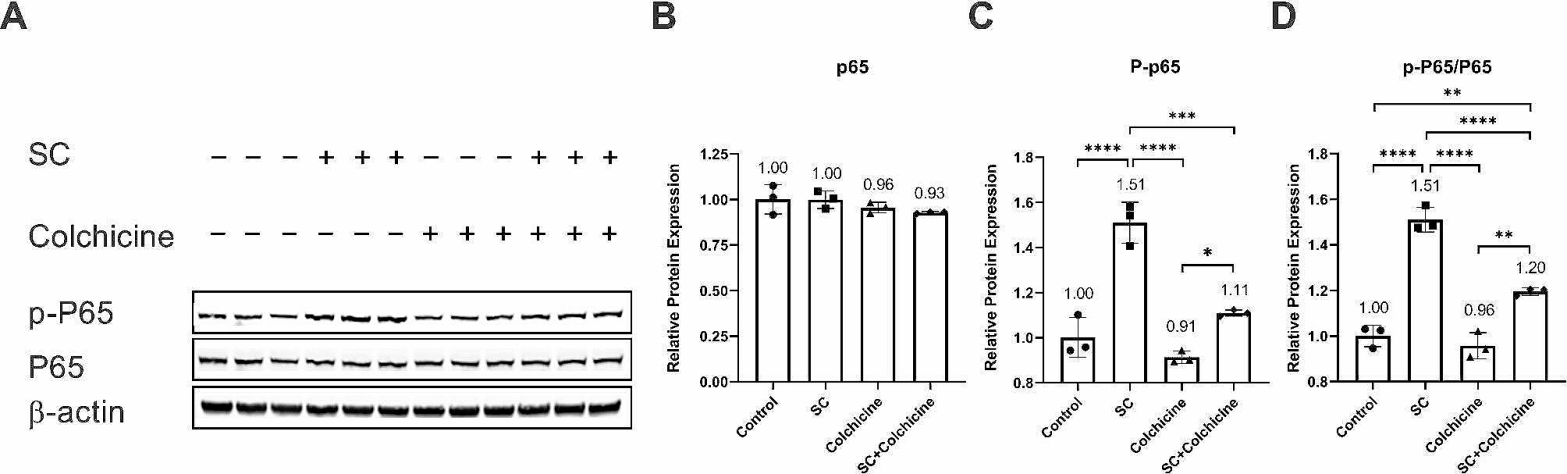
Colchicine inhibited NF-κB activation in tobacco smoke-condensate-treated endothelial cells. (**A**) WB showing protein expression and phosphorylation of NF-κB subunit P65. (**B**) The quantification of the relative protein expression of NF-κB subunit P65 and (**C**) phosphorylated NF-κB subunit P65. (**D**) The ratio of the relative protein expression of phosphorylated NF-κB subunit P65 and the relative protein expression of NF-κB subunit P65. The experiments were performed with biological triplicates. β-actin was used as a control. *p < 0.05, **p < 0.01, ***p < 0.001, ****p < 0.0001

The activation of NF-kB, MAPKs, and mTOR pathways contributes to senescence and the expression and release of SASP factors [15]. We performed protein analysis to investigate the activation of these pathways in endothelial cells treated with 50 µg/mL Tobacco smoke condensate, 50 nM colchicine, and 50 µg/mL tobacco smoke condensate combined with 50 nM colchicine for 24 h. The untreated endothelial cells were used as a control. Neither of the treatments affected the relative protein expression of NF-κB subunit P65 (Fig. 4A, B; Table 1). Tobacco smoke condensate increased the phosphorylation of NF-κB subunit P65, and colchicine attenuated NF-κB phosphorylation in endothelial cells treated with tobacco smoke condensate (Fig. 4A, C, D; Table 1).

**Fig. 5:**
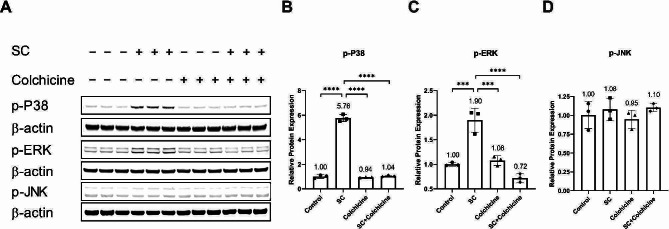
Colchicine prevented the activation of P38 and ERK. (**A**) WB showing protein expression of MAPKs, p-P38, p-ERK, and p-JNK. The quantification of relative protein expression of (**B**) p-P38, (**C**) p-ERK, and (**D**) p-JNK in endothelial cells treated with different conditions. The experiments were performed with biological triplicates. β-actin was used as a control. ***p < 0.001, ****p < 0.0001

Colchicine reduced the relative protein expression of the phosphorylated P38 and ERK in tobacco smoke-condensate-treated endothelial cells (Fig. 5A, B, C; Table 1). Neither treatment affected the relative protein expression of p-JNK (Fig. 5A, D; Table 1).

**Fig. 6:**
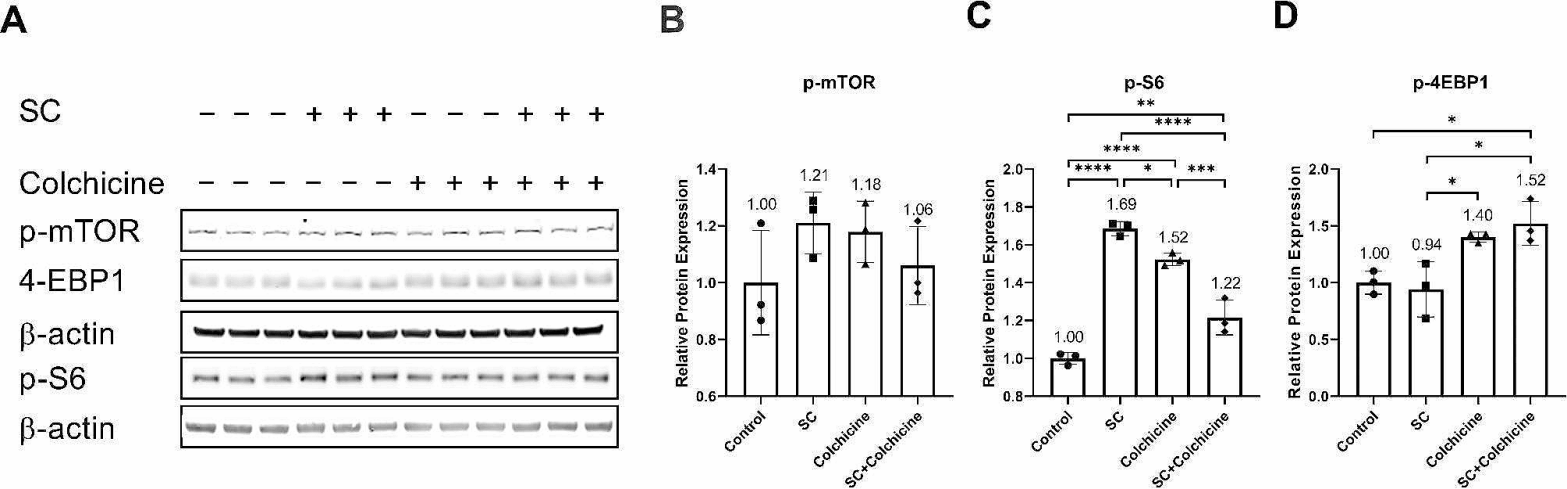
Colchicine modulates mTOR pathway activation. (**A**) WB showing protein expression of p-mTOR, p-S6, and p-4EBP1. The quantification of the relative protein expression of (**B**) p-mTOR, (**C**) p-S6, and (**D**) p-4EBP1 in endothelial cells treated with different conditions. The experiments were performed with biological triplicates. β-actin was used as a control. *p < 0.05, **p < 0.01, ***p < 0.001, ****p < 0.0001

Neither treatment significantly affected the relative protein expression of p-mTOR (Fig. 6A, B; Table 1). Both smoke condensate and colchicine increased the relative protein expression of p-S6 (Fig. 6A, C; Table 1). The relative protein expression of p-S6 is lower in endothelial cells treated with tobacco smoke condensate combined with colchicine than in the endothelial cells treated with either tobacco smoke condensate or colchicine (Fig. 6A, C; Table 1). The relative protein expression of p-4EBP1 was significantly higher in colchicine-treated endothelial cells than in tobacco smoke-condensate-treated endothelial cells (Fig. 6A, D; Table 1). The relative protein expression of p-4EBP1 was significantly elevated in endothelial cells treated with tobacco smoke condensate combined with colchicine compared to control and endothelial cells treated with smoke condensate (Fig. 6A, D; Table 1).

## Discussion

Hitherto, state-of-the-art suggests that smoking is a causative agent for many diseases, including CVDs and cancer [1–3]. Smoke condensate generated oxidative stress, induced DNA damage, caused endothelial cell senescence, activated NF-κB and MAPKs, and increased the transcription of SASP factors. Here, we discover colchicine to alleviate the damaging effect of tobacco smoke condensate on endothelial cells.

Smoking begets oxidative stress, resulting in oxidative stress-induced DNA damage, and thus can cause cellular senescence [4–6]. Colchicine mitigated oxidative stress (Fig. 1A, B) and oxidative stress-induced DNA damage (Fig. 1C, D) in endothelial cells treated with tobacco smoke condensate (Fig. 1B, D). Previously, colchicine has been shown to provide anti-oxidative benefits [24, 25, 30], attenuate oxidative stress-induced DNA damage, impede pyroptosis and cellular senescence in endothelial cells [24, 26, 27]. Interestingly, tobacco smoke condensate increased the relative protein expression of DNA repair proteins KU70/KU80 (Fig. 1F, G), which was mitigated by colchicine (Fig. 1F, G), contrary to previously reported findings, where colchicine improved the relative protein expression of KU70/KU80, which was decreased in endothelial cells treated with H_2_O_2_ and ethanol [26, 27]. KU70/KU80 form a heterodimer and repair double-strand DNA breaks [31]. The expression of KU70/KU80 (Fig. 1F, G) might have increased in response to DNA damage (Fig. 1C, D) in endothelial cells exposed to tobacco smoke condensate. However, as colchicine diminished oxidative stress and oxidative stress induced DNA damage (Fig. 1A, B, C, D) in endothelial cells exposed to tobacco smoke condensate, the expression of KU70/KU80 (Fig. 1F, G) returned to control levels.

Cellular senescence is a complex biological process. The exhibition of multiple characteristics (categorized into three groups) by cells has been suggested to confirm cellular senescence [7, 8]. Briefly, senescent cells display (a) structural change, (b) growth arrest, and (c) secondary markers like enhanced expression and release of SASP factors [7, 8]. To validate a structural change in tobacco smoke-condensate-treated endothelial cells, we performed SA-β-gal staining and quantified the protein expression of Lamin B1 by doing IF staining and WB. Tobacco smoke condensate increased SA-β-gal activity (Fig. 2A, C) and lowered the expression of Lamin B1 (Fig. 2B, D, F, and Table 1) in endothelial cells. Colchicine lessened SA-β-gal activity (Fig. 2A, C) and rescued Lamin B1 expression in endothelial cells exposed to tobacco smoke condensate (Fig. 2B, D, F, and Table 1), confirming the previously reported findings, which have shown that colchicine diminished SA-β-gal activity and improved Lamin B1 expression in endothelial cells exposed to ethanol and oxidative stress [26, 27]. Lamin B1 maintains nuclear stability and regulates DNA replication and gene transcription [32]. Lamin B1 loss can induce premature senescence [33]. During Senescence, Lamin B1 expression diminishes because of reduced Lamin B1 mRNA stability [34]. Furthermore, Lamin B1 loss can activate the P53-P21 cell cycle arrest pathway [33].

The cell cycle arrest in endothelial cells exposed to tobacco smoke condensate was confirmed by the increased relative protein expression of cell cycle arrest markers P53 (Fig. 2G) and P21 (Fig. 2H) in endothelial cells. DNA damage can rapidly increase the levels of P53 and stabilize it [6]. Increased P53 suppresses the transcription of cell cycle genes, and this indirect repression of cell cycle genes by P53 is mediated through P21 [6, 35, 36]. P53 induces P21 expression [36, 37], which results in the formation of the RB-E2F complex that downregulates genes involved in the cell cycle, leading to cell cycle arrest and senescence [6, 36]. P53 can also cause Lamin B1 loss [34]. These findings suggest that by mitigating P53 (Fig. 2G; Table 1) and P21 (Fig. 2H, I; Table 1), colchicine restored cell cycle growth in endothelial cells treated with tobacco smoke condensate.

The senescence-associated secretory phenotype is the secondary marker of cellular senescence [7, 8]. The senescent cells increase the expression and release of SASP factors [7, 8]. We quantified the transcription of SASP factors in endothelial cells. Tobacco smoke condensate enhanced the mRNA expression of SASP factors in endothelial cells (Fig. 3). The expression of SASP factor is increased in endothelial cells as a consequence of stress induced premature senescence [26, 27, 38, 39]. Colchicine mitigated the mRNA expression of the SASP factors induced by tobacco smoke condensate in endothelial cells (Fig. 3), confirming the previously reported findings, where colchicine suppressed the mRNA expression of SASP factors in endothelial cells treated with ethanol and oxidative stress [26, 27]. These findings suggest that colchicine inhibited senescence (Figs. 2 and 3; Table 2) in endothelial cells treated with smoke condensate.

Selective elimination of senescent cells reversed atherosclerosis, enhanced stability and improved vulnerability of atherosclerotic plaques, reduced fibrosis, alleviated cardiomyocyte hypertrophy, and increased heart regenerative capacity [14–16]. In the context of cancer, the targeted removal of senescent cells alleviated the adverse effects of cancer therapy and lowered the risk of cancer progression [11]. The elevated expression and release of SASP factors contribute to the initiation and progression of age-related diseases [40, 41]. SASP factors play contrary roles in the initiation and progression of cancer [11]. On the one hand, the enhanced expression of SASP factors via activating immune response can remove cancer cells and promote cell growth arrest [11, 42]. On the other hand, the chronic SASP can potentially promote cancer by contributing to metastasis, angiogenesis, invasiveness, and tumorigenesis in the neighboring cells [11, 42]. Colchicine ameliorated the expression of SASP factors, including IL-6, MCP-1, ICAM-1, E-Selectin, MMP-2, MMP-11, and TIMP-1 (Fig. 3; Table 2) in endothelial cells treated with tobacco smoke condensate. Previous studies have shown that colchicine in vivo and in vitro reduced the expression of IL-1β, IL-6, IL-8, IL-18, TNF-α, MCP-1, ICAM-1, VCAM-1, and E-selectin at mRNA and protein level [23–27]. SASP factors such as IL-6 promote chronic sterile inflammation, which is known to promote the initiation and progression of CVDs and support cancer growth and invasiveness [15, 17, 42]. Clinical and experimental studies implicate IL-6 in the progression of cancers [11] and CVDs, including hypertension, atherosclerosis, aortic and thoracic aneurysms, cardiac fibrosis, aortic dissection, cardiomyopathy, heart failure, and ischemic stroke [40, 41]. SASP factors such as IL-1β, IL-8, TNF-α, and MCP-1 recruit immune cells, including macrophages, neutrophils, and T-cells [15, 17]. ICAM-1, E-selectin, and MMPs promote tissue infiltration and accumulation of inflammatory cells [15, 17]. The accumulation of inflammatory cells exacerbates local inflammation and contributes heavily to CVD and Cancer [15, 17]. Colchicine attenuated the activation of circulating monocytes in vitro [22] and reduced the adhesion of monocytes to HUVECs [25]. In experimental animal studies, colchicine dampened the accumulation of inflammatory cells in tissue [22, 23] and protected against CVDs, including dilated cardiomyopathy, atherosclerosis, and thrombogenesis [22, 23, 25]. Furthermore, MMPs contribute to CVDs and cancers by degenerating extracellular matrix and mediating inflammation through post-translational processing of inflammatory cytokines, chemokines, cell adhesion molecules, and their receptors on cells [18, 42]. Interestingly, colchicine reduced MMP-2 and MMP-11 expression but did not reduce TIMP2 expression. TIMP2 inhibits MMP-1, MMP-2, MMP-8, and MMP-11, suggesting colchicine reducing the expression of some MMPs and keeping TIMP2 expression high can suppress MMPs mediated tissue modulation and inflammation [18]. Therefore, it can be postulated that through mitigating the expression of SASP factors (Fig. 3; Table 2) [23–27], colchicine can suppress the initiation and progression of CVDs [22, 23, 25] and metastasis, angiogenesis, and growth in cancers [42].

Our pathway analysis showed that tobacco smoke condensate activated NF-κB (Fig. 4) and MAPKs P38 and ERK (Fig. 5). It has already been reported that DNA damage, oxidative stress, and ethanol can activate these pathways [21, 26, 27, 38, 39]. Activation of these pathways has been linked to cellular senescence, and inhibiting these pathways could block cellular senescence [21]. After activation, these pathways enhance the transcription, protein expression, and stability of senescence-driving proteins such as P53 and P21 [21, 37, 43, 44]. Moreover, MAPKs P38 and ERK increase the levels of SASP factors through NF-κB transcriptional activity [21]. The activation of P38 is needed for NF-κB transcriptional activity in senescence [45]. Interestingly, colchicine, without significantly affecting p-mTOR expression, increased the relative protein expression of mTOR downstream signalling molecules p-S6 and p-4EBP-1 (Fig. 6). These two downstream signalling molecules have the opposite effect on life span [46–48]. S6K deficiency in mice and flies extended mean life span [46, 48] and 4EBP-1 overexpression in flies increased life span [47]. Therefore, the net effect of colchicine through mTOR pathway activation would be challenging to determine. Taken together, it is very likely that by blocking NF-κB, P38- and ERK (Figs. 4 and 5) [15, 21, 26, 27, 44], colchicine suppressed senescence and the expression of SASP factors in endothelial cells (Figs. 2 and 3) treated with tobacco smoke condensate.

## Conclusion

Tobacco smoking is one of the potential acquired causative risk factors for chronic and age-related diseases. Colchicine prevented tobacco smoke condensate-induced DNA damage and senescence in endothelial cells exposed to smoke condensate. It mitigated the expression of SASP factors in endothelial cells treated with tobacco smoke condensate. Colchicine blocked tobacco smoke-condensate-induced activation of NF-κB, P38, and ERK. These findings suggest that by suppressing the activation of NF-κB and MAPKs, colchicine inhibited senescence in endothelial cells treated with tobacco smoke condensate. The current findings will have implications in cardiovascular diseases.

## Materials and methods

### Cell Culture

Three different HUVEC models were procured from Promocell (Heidelberg, Germany). Endothelial cell medium (C-22,010, Promocell, Heidelberg, Germany) containing endothelial growth factors (C-39,215, Promocell, Heidelberg, Germany) was used to maintain endothelial cells at 37 °C in a humidified environment at 37 °C and 5% CO2. Upon arrival, the cells were thawed and seeded in T75 culture flasks. The cells were passaged when they reached 80–90% confluence. For passaging, the cells were washed with PBS and incubated with trypsin for 4 min at 37 °C in a humidified environment at 37 °C and 5% CO2. The cells were seeded at a density of 5000 cells/cm^2^ in new cell culture plates. All experiments were performed at passage 7. The cells were treated with either 50 µg tobacco smoke condensate, 50 nm colchicine or tobacco smoke condensate combined with colchicine. Untreated cells were used as control. All experiments were performed with three biological replicates except Lamin B1 and DCFH-DA staining, where one HUVEC cell model was used.

### Tobacco smoke condensate preparation

Commercially available cigarettes were smoked through ethanol. After that, ethanol was evaporated at room temperature. Tobacco smoke condensate was weighed, and 100 mg/mL of tobacco smoke condensate was dissolved in DMSO.

### DCFH-DA staining

To investigate the accumulation of cellular ROS, 5000 cells/cm^2^ endothelial cells were seeded in a 96-well plate. The next day, the medium was changed with a new medium either containing 50 µg/mL tobacco smoke condensate, 50 nM colchicine, or 50 µg/mL smoke condensate combined with 50 nM colchicine. Endothelial cell medium alone was used as a control. After 2 h of treatment, 10 µM of the fluorescence probe 2,7-dichlorofluorescein diacetate (DCFH-DA, D6883, Sigma-Aldrich, MO, USA) was added to the cells. The cells were incubated with DCFG-DA at 37 °C for 30 min in the dark. The cells were washed three times with a serum-free medium. The images were taken with a fluorescence microscope and analyzed with image J.

### Immunofluorescence staining

The cells were treated with different conditions (as described in the previous section) for 2 h for 8-OHDG staining and 24 h for Lamin B1 staining. After washing cells thrice with PBS, the cells were fixed with 4% paraformaldehyde for 10 min at RT. After washing cells thrice with PBS, for permeabilization, the cells were treated with 0.2% Triton™ X-100 at RT for 10 min. For blocking, the cells were incubated with 5% bovine serum albumin (BSA) at RT for 1 h. The cells were incubated overnight with primary antibodies 8-OHDG (Supplementary table S1) and Lamin B1 (Supplementary table S1) at 4° C. The next day, after washing cells thrice with PBS, the cells were incubated with secondary antibodies (Supplementary table S1) for 1 h at RT. HOECHST (Sigma-Aldrich) was used for nuclear staining. The images were captured using a Leica DMi8 Inverted Microscope and the compatible LAS-X Life Science Microscope Software (Leica Application Suite X) Platform. The images were analyzed using ImageJ (version 1.53c) (National Institutes of Health, Bethesda, MD, USA).

### Western blot

For protein analysis, HUVECs were treated with 50 µg/mL smoke condensate, 50 nM colchicine, or 50 µg/mL smoke condensate combined with 50 nM colchicine for 24 h. Untreated endothelial cells were used as controls. RIPA buffer was used for total protein extraction. DC Protein Assay Kit (500–0116, Bio-Rad, Hercules, CA, USA) was used to quantify protein concentration. Subsequently, 30 µg of total protein under reducing conditions was loaded onto a 12% sodium dodecyl sulfate-polyacrylamide gel. For the first 20 min, electrophoresis was conducted at 60 Volts, followed by 110 Volts for 30–60 min. The separated proteins were then transferred onto a 0.45 μm pore –size nitrocellulose membrane at 250 mA for 120 min. The membranes were blocked for one hour with a 5% bovine serum albumin (BSA) solution in 0.05% TBST to minimize nonspecific binding. After that, the membranes were incubated with primary antibodies (see Supplementary Table S1) in 5% BSA overnight at 4 °C on a shaking platform. Afterward, the membranes underwent 3 × 10 min washes with TBST and were subsequently exposed to secondary antibodies diluted in 0.05% TBST (refer to Supplementary Table S1) for one hour at room temperature. Densitometry analysis was performed using NIHImageJ with β-actin correction.

### Quantitative polymerase chain reaction (qPCR)

For qPCR analysis, the endothelial cells were treated as described in the previous section. Total RNA was extracted using the Nucleo Spin RNA kit (740955.50, MACHEREY-NAGEL, Düren, Germany) according to the manufacturer’s instructions. A total of 1.2 µg of RNA was utilized for reverse transcription, accomplished using the MMLV Reverse Transcriptase kit (M1701, Promega, Walldorf, Germany), Random Hexamer Primers (48,190,011, Thermo Fisher), and RiboLock RNase Inhibitor (EO0384, Thermo Fisher). The qPCR was run using total cDNA combined with AceQ SYBR qPCR Master Mix (Q111-03, Vayzme, Nanjing, China) and primers (Supplementary Table 2) on a Bio-Rad thermal cycler. The thermal cycling program consisted of an initial denaturation step at 95 °C for 8 min, followed by 40 cycles of 95 °C for 15 s, 58.9 °C for 30 s, and 72 °C for 30 s, concluding with a melting curve analysis. To calculate relative mRNA expressions, data were normalized to β-actin expression, and the relative expression levels were quantified using the comparative ΔCT method.

### Senescence associated β-Galactosidase staining

For senescence associate beta-galactosidase (SA-β-Gal) staining, endothelial cells were treated for 24 h as described in the previous sections. SA β-Gal staining was performed using the Senescence Cells Histochemical Staining Kit (GALS, Sigma, MO, USA) following the manufacturer’s instructions. The cells were incubated with SA–-β-galactosidase staining solution at 37 °C for seven hours. The staining solution was aspirated and the cells were overlaid with 70% glycerol in PBS. After staining, the cells were stored at 4 °C. The images were captured using a Leica DMi8 Inverted Microscope and the compatible LAS-X Life Science Microscope Software (Leica Application Suite X) Platform. The images were analyzed using ImageJ (version 1.53c) (National Institutes of Health, Bethesda, MD, USA).

### Statistical analysis

We analyzed the data on PRISM using one-way ANOVA followed by Tukey’s post hoc test.

### Electronic supplementary material

Below is the link to the electronic supplementary material.

Supplementary Material 1

## Data Availability

The datasets supporting the conclusions of this article are included within the article and its additional file.
