# Supplementary material for: Tobacco smoke condensate-induced senescence in endothelial cells was ameliorated by colchicine treatment via suppression of NF-κB and MAPKs P38 and ERK pathways activation
Source: Cell Commun Signal. 2024 Apr 3;22:214. doi: 10.1186/s12964-024-01594-x (PMC10988825; doi:10.1186/s12964-024-01594-x)
Supplement: Supplementary file 1 — Supplementary Material 1 [file 12964_2024_1594_MOESM1_ESM.docx]

**Table 1.** Primary and Secondary antibodies

| Antibody | MW(kDa) | Brand | Catalog Number | Concentration |
| --- | --- | --- | --- | --- |
| P53 | 53 | Abcam | #ab28 | 1:1000 |
| P21 | 21 | Cell Signaling | # 2947S | 1:1,000 |
| Lamin B1 | 66 | Abcam | ab16048 | 0.1 μg/mL |
| KU80 | 86 | Cell Signaling | #2753S | 1:1,000 |
| Ku70 | 70 | Cell Signaling | #4588S | 1:1,000 |
| P65 | 65 | Cell Signaling | #6956S | 1:1,000 |
| p-P65 | 65 | Cell Signaling | #3033S | 1:1,000 |
| MMP-2 | 70 | Invitrogen | 436000 | 2 μg/mL |
| β-actin (Rabbit) | 45 | Cell Signaling | #4970S | 1:1,000 |
| β-actin (Mouse) | 45 | Invitrogen | #MA5-15739 | 1:1,000 |
| p-P38 | 43 | Cell Signaling | #4511S | 1:1,000 |
| p-JNK | 46,54 | Cell Signaling | #9255S | 1:2,000 |
| p-ERK | 42,44 | Cell Signaling | #4370S | 1:2,000 |
| p-mTOR | 289 | Cell Signaling | #2971S | 1:1,000 |
| p-S6 | 32 | Cell Signaling | #2215S | 1:1,000 |
| p-4EBP1 | 15-20 | Cell Signaling | #2855S | 1:1,000 |
| 8-OHDG |  | BIOSS | BSS-BS-1278R | 1:500 |
| Alexa Fluor Plus 488 |  | Abcam | ab150077 | 1:10,000 |
| IRDye 800CW Goat-anti-Rabbit Antibody |  | LiCor | #926-32211 | 1:10,000 |
| IRDye 680RD Donkey anti-Mouse IgG Secondary Antibody |  | LiCor | #926-68072 | 1:10,000 |

**Table 2.** Primer list

| Target gene | Gene Accession Number | Sense 5’ -3’ | Antisense 5’ -3’ |
| --- | --- | --- | --- |
| ICAM-1 | NM_000201 | CACAGTCACCTATGGCAACGA | TGGCTTCGTCAGAATCACGTT |
| VCAM-1 | NM_080682,  NM_001199834,  NM_001078 | AGTGGTGGCCTCCTGAATGG | CTGTGTCTCCTGTCTCCGCT |
| IL-8 | NM_001354840,  NM_000584 | TGCCAAGGAGTGCTAAAG | CTCCACAACCCTCTGCAC |
| MCP-1 | NM_002982 | CACCAATAGGAAGATCTCAGTGC | TGAGTGTTCAAGTCTTCGGAGTT |
| MMP1 | NM_001145938,  NM_002421 | CAGAGATGAAGTCCGGTTTTTC | GGGGTATCCGTGTAGCACAT |
| MMP2 | NM_001302510,  NM_001302509,  NM_001127891,  NM_004530,  NM_001302508 | ATAACCTGGATGCCGTCGT | AGGCACCCTTGAAGAAGTAGC |
| MMP8 | NM_001304442, NM_001304441, NM_002424 | TGGGGCTCGCTCACTCCTC | ATCAAATGTCAAACTGGGGTCAC |
| MMP10 | NM_002425 | CACAGTTTGGCTCATGCCTA | TGCCATTCACATCATCTTGC |
| MMP11 | NM_005940 | CCGCAACCGACAGAAGAGG | ATCGCTCCATACCTTTAGGGC |
| TIMP1 | NM_003254 | TGGCTTCTGGCATCCTGTTGTTG | CGCTGGTATAAGGTGGTCTGGTTG |
| TIMP2 | NM_003255 | GAATCGGTGAGGTCCTGTCCTGA | CCTGCACACAAGCCCGGATAAA |
| IL-1β | NM_000576 | AGATGATAAGCCCACTCTACAG | ACATTCAGCACAGGACTCTC |
| E-selectin | NM_000450 | CAAGAAGAAGCTTGCCCTATG | ACTTGAGTCCACTGAAGCCA |
| IL-6 | NM_001371096  NM_001318095 XM_011515391  NM_000600 | CTGCAGGACATGACAACTCATC | ATCTGAGGTGCCCATGCTAC |
| P21 | NM_001374511, NM_001220777, NM_001374510, NM_001374512, NM_001374513, NM_001291549, NM_078467, NM_001374509, NM_001220778, NM_000389 | GACACCACTGGAGGGTGACT | CAGGTCCACATGGTCTTCCT |
| TNF-α | NM_000594 | CCCGAGTGACAAGCCTGTAG | GATGGCAGAGAGGAGGTTGAC |
